# Reply to: “Methodological considerations on purpose in life of older adults”

**DOI:** 10.1055/s-0046-1829020

**Published:** 2026-11-03

**Authors:** Sabrina Aparecida da Silva, Gabriela dos Santos, Tiago Nascimento Ordonez, Wellington Lourenço Oliveira, Diana dos Santos Bacelar, Maria Antonia Antunes de Souza, Laydiane Alves Costa, Henrique Salmazo da Silva, Beatriz Aparecida Ozello Gutierrez, Rosa Yuka Sato Chubaci, Sonia Maria Dozzi Brucki, Thais Bento Lima da Silva

**Affiliations:** 1 Universidade de São Paulo, Escola de Artes, Ciências e Humanidades, Programa de Pós-Gradfuação em Gerontologia, São Paulo SP, Brazil.; 2 Universidade Federal do Recôncavo da Bahia, Centro de Ciências da Saúde, Santo Antônio de Jesus BA, Brazil.; 3 Universidade de São Paulo, Faculdade de Medicina, Hospital das Clínicas, Departamento de Neurologia, São Paulo SP, Brazil.

Dear Editor,

We thank Jaiswal and Saxena 1 for their insightful comments on our article. Their observations offer a valuable opportunity to discuss the methodological nuances involved in the assessment of purpose in life (PiL) in older adults.

Regarding the study population, 2 we acknowledge that participants drawn from the 24-month follow-up of a cognitive stimulation trial may introduce selection bias. However, the original intervention was not specifically designed to focus on existential or motivational constructs such as PiL, and we deliberately included participants from all original trial arms to reduce the likelihood of trial-related selection factors systematically influencing our cross-sectional analysis of PiL. We agree that future studies would benefit from adjustment for original trial allocation or stratification by treatment arm to further strengthen internal validity.

With respect to the dichotomization of PiL scores and our use of a stepwise logistic regression model, these approaches were chosen because the continuous and ordinal variables did not follow a normal distribution. Although this method allowed us to identify the most relevant predictors in our dataset using the Mann-Whitney U test, Spearman correlation, and logistic regression, we agree with the authors 1 that keeping PiL as a continuous variable in generalized linear models could preserve data variability and statistical power, and this should be strongly considered in future studies.

We also agree with the importance of interpreting effect sizes alongside statistical significance. In the discussion section, we took care to emphasize that the associations observed were weak, indicating that sociodemographic factors alone explain only limited variation in PiL. The findings are consistent with those of the international literature: AshaRani et al., 3 in a systematic review of 44 studies, found female sex, higher education, and higher income to be sociodemographic determinants of PiL in older adults, although factors such as wellbeing, inner strength, social integration, and spirituality also play important roles. Similarly, Steptoe and Fancourt, 4 in a study of 7,304 participants aged 50 years and older, showed that greater educational level was associated with higher levels of purpose in life, but that stronger personal relationships, greater social engagement, lower loneliness, better mental and physical health, and healthier lifestyles were strongly associated with the construct.

Additionally, Coelho et al., 5 in a study of 1,330 Portuguese adults, showed that education, spirituality, optimism, social support, and physical health-related quality of life contributed positively to PiL, reinforcing the multifactorial nature of this construct. Thus, we explicitly acknowledge in our limitations that crucial determinants such as subclinical depressive symptoms, social support, physical health, functional status, and religiosity were not assessed and likely represent a substantial proportion of the unexplained variance.

With respect to administration of the PiL scale by telephone, rigor was ensured by reading the standardized instructions literally ( *verbatim* ) to all participants. In addition, the study sample had previously completed the Brazilian version of the Mini-Mental State Examination specifically validated for telephone administration (Braztel-MMSE), demonstrating the feasibility of remote assessments in this group. Nevertheless, we agree that providing estimates of internal consistency for the PiL scale administered by telephone remains an important recommendation to support the reliability of remote measurement.

Lastly, regarding the title of the article, we transparently reported in the Conclusion of our Abstract and in the Discussion that, although age showed a weak negative bivariate correlation with PiL, it did not remain an independent predictor in the final multivariable model. We appreciate the suggestion for more precise alignment of the title in future work to avoid overinterpretation. We also thank the authors for their constructive feedback, which undoubtedly enriches the scientific discussion on healthy aging in the context of a low- and middle-income country.
